# Antioxidant Activity and Genotoxic Assessment of Crabwood (Andiroba, *Carapa guianensis* Aublet) Seed Oils

**DOI:** 10.1155/2018/3246719

**Published:** 2018-05-02

**Authors:** Carlos F. Araujo-Lima, Andreia S. Fernandes, Erika M. Gomes, Larisse L. Oliveira, Andrea F. Macedo, Rosemar Antoniassi, Allan E. Wilhelm, Claudia A. F. Aiub, Israel Felzenszwalb

**Affiliations:** ^1^Environmental Mutagenesis Laboratory, Department of Biophysics and Biometry, Rio de Janeiro State University (UERJ), Rio de Janeiro, RJ, Brazil; ^2^Genotoxicity Laboratory, Department of Genetics and Molecular Biology, Federal University of the State of Rio de Janeiro (UNIRIO), Rio de Janeiro, RJ, Brazil; ^3^Integrated Laboratory of Plant Biology, Department of Botany, Institute of Biosciences, Federal University of State of Rio de Janeiro, Rio de Janeiro, RJ, Brazil; ^4^Embrapa Food Technology, Brasília, DF, Brazil

## Abstract

The seed oil of *Carapa guianensis* (Aublet), a tree from the Meliaceae family commonly known as andiroba, is widely used in Brazilian traditional medicine because of its multiple curative properties against fever and rheumatism and as an anti-inflammatory agent, antibacterial agent, and insect repellant. Since there is no consensus on the best way to obtain the *C. guianensis* oil and due to its ethnomedicinal properties, the aim of the present research was to evaluate the chemical composition, free-radical scavenging activity, and mutagenic and genotoxicity properties of three *C. guianensis* oils obtained by different extraction methods. The phenolic contents were evaluated by spectrophotometry. Oil 1 was obtained by pressing the dried seeds at room temperature; oil 2 was obtained by autoclaving, drying, and pressing; oil 3 was obtained by Soxhlet extraction at 30–60°C using petroleum ether. The oil from each process presented differential yields, physicochemical properties, and phenolic contents. Oil 1 showed a higher scavenging activity against the DPPH radical when compared to oils 2 and 3, suggesting a significant antioxidant activity. All oils were shown to be cytotoxic to bacteria and to CHO-K1 and RAW264.7 cells. At noncytotoxic concentrations, oil 2 presented mutagenicity to *Salmonella enterica* serovar Typhimurium and induced micronuclei in both cell types. Under the same conditions, oil 3 also induced micronucleus formation. However, the present data demonstrated that oil 1, extracted without using high temperatures, was the safest for use as compared to the other two oils, not showing mutagenicity or micronucleus induction.

## 1. Introduction

*Carapa guianensis* (Aublet) is a large neotropical tree belonging to the Meliaceae family. It can be found in the north of South America, Central America, the Caribbean, and Sub-Saharan Africa. In Brazil, it is known as andiroba occurring mainly in lowlands and flooded areas throughout the Amazon region [1, 2].

Some studies have reported several activities produced by the seed oil from *C. guianensis* and used in folk medicine, such as treating fever and rheumatism, antiallergic, analgesic, chemotherapeutic, and anti-inflammatory effects [3–7], as well as acaricidal and insect repellent action [8–10]. It is also effective against rheumatism and arthritis [1]. Furthermore, the infusion prepared with the bark and flowers of *C. guianensis* is used both as an anthelmintic and wound-healing agent in humans [11].

There is no consensus on the best way to obtain *C. guianensis* seed oil. Traditional techniques are time-consuming and may include cooking, drying in the sun, and allowing enzymatic action and fermentation [12, 13], and the mechanical extraction of chopped seeds, including drying and pressing at around 90°C, has also been used [14].

These processes generate a large quantity of residual seed material as a by-product, which contains many bioactive constituents including limonoids or tetranortriterpenoids [9]. The chemical components of *C. guianensis* seeds have been extensively studied over the years, mainly gedunin-type limonoids bearing the 4,4,8-trimethyl-17-furanylsteroid. Limonoids present several biological activities, including antifungal and bactericidal activities and a variety of medical effects in animals and humans [15].

Crude vegetable oils or fixed oils are composed mainly of triacylglycerols (around 95%) along with some free acids, monoacylglycerols, and diacylglycerols. They also contain variable amounts of other components such as phospholipids, free and esterified sterols, triterpene alcohols, tocopherols and tocotrienols, phenolic compounds, carotenes, chlorophylls, hydrocarbons, oxidation compounds, metals, and minor and trace compounds. The amounts of each of these compounds depend on the extraction and oil-refining processes [16].

The biological activity of fixed oils has been associated with the fatty acids or their glycerides, to different lipid classes (such as phospholipids and glycolipids) and to a plethora of minor compounds present in the oils [17].

According to the Organization for Economic Cooperation and Development [18], all products intended for human use must be evaluated regarding toxicological aspects. In general, the first toxicological evaluation of new chemicals is the *Salmonella*/microsome reverse mutation assay, which detects punctual mutations in the DNA sequence, followed by an evaluation of the clastogenicity potential using the micronucleus assay [19].

Thus, the aim of the present study was to evaluate the chemical composition, the free radical scavenging activity, and the mutagenic and genotoxicity properties of *C. guianensis* oils obtained via three different extraction methods. Phenolic contents were evaluated by spectrophotometry.

## 2. Methods

### 2.1. *C. guianensis* Oil Preparation

The *C. guianensis* Aublet C. seeds were harvested from December 2013 to February 2014 from under identified parent trees in the Rio de Janeiro Botanical Gardens, with prior authorization. The seeds were superficially cleaned and frozen at −18°C, and for oil extraction, the frozen seeds were first sliced and air-dried at 60°C.

After preparing the sliced and dried seeds, oil 1 was obtained by pressing using a model CA59G expeller-type laboratory press (Komet) with a maximum processing capacity of 5 kg/h of raw material. Screws with 12 mm between the grooves were used, with an oil outlet grid with 1 mm diameter holes and a 10 mm diameter outlet nozzle. The oil obtained was centrifuged at 10,000 rpm for 10 minutes at room temperature to remove particles and then frozen at −18°C.

To evaluate the effects of the thermal treatment, the frozen seeds were autoclaved at 121°C for 15 min and then submitted to slicing, drying, and pressing conditions as presented above to obtain oil 2.

Oil 3 was obtained using the Soxhlet extraction method (30–60°C for 16 h) with petroleum ether, extracting from the dried seeds with no further heat treatment. The solvent was evaporated off in a rotary evaporator under a stream of nitrogen, and the oil obtained was frozen at −18°C.

### 2.2. Fatty Acid Composition

In order to analyze the fatty acid profile, methyl esters were obtained according to Hartman and Lago [20]. Gas chromatography was carried out using an Agilent 7890 gas chromatograph with a fused silica capillary column (60 m; 0.32 mm internal diameter; and 0.25 *μ*m stationary phase of 78% cyanopropyl methylpolysiloxane) with a temperature program from 150 to 200°C at a rate of 1.3°C/min.

The injector temperature was 250°C maintaining the injector in the split flow mode with a ratio of 50 : 1 and injecting 1 *μ*L of the oil diluted in dichloromethane (2%). The temperature of the flame ionization detector was 280°C, and the carrier gas flow (H_2_) was 2.5 mL/min (measured at 40°C). The fatty acid methyl esters were identified by a comparison of their retention times with standard Nu Chek (Elysian, MN) numbers 62, 79, and 87 and quantified by an internal normalization. The iodine and saponification values were calculated from the fatty acid profile.

### 2.3. Physicochemical Aspects

The free fatty acid content of the oils was determined by titration according to the American Oil Chemists' Society official method number Ca 5a 40 (AOCS) (2009) [21], and the results were expressed as oleic acid. The peroxide content (milliequivalents of peroxide/1000 g of sample) was determined according to the AOCS method number Cd 8 53 (2009). The refractive index was measured in a Bausch and Lomb Abbé refractometer at 40°C and the density in a PAAR DMA AP-46 digital densitometer at 20°C.

### 2.4. DPPH Assay

DPPH (2,2-diphenyl-1-picrylhydrazyl) radical scavenging was evaluated according to a previously reported procedure [22] with some modifications [23]. The DPPH solution was freshly prepared daily, stored in a flask, covered, and kept in the dark at 4°C. Briefly, one milliliter of 0.1 mM DPPH in absolute methanol was added to one milliliter of each sample dilution, with five concentration levels ranging from 0 to 200 *μ*L dissolved in dimethyl sulfoxide (DMSO). The solutions were mixed, covered, and allowed to react in the dark at room temperature for 30 min at 25°C, and the absorbance was measured at 517 nm in a spectrophotometer (Shimadzu UV-160A spectrophotometer). The control was prepared by mixing the DPPH-methanol solution with the sample solvent or butylated hydroxytoluene (BHT, positive control). The blank was prepared with ethanol plus the extract solution. The DPPH radical scavenging activity of the sample was calculated according to the following equation: %inhibition = (control − sample)/(control − blank) × 100%. The EC_50_ values were calculated by an interpolation from the linear regression analysis.

### 2.5. Total Phenolic Content

The total phenolic content was estimated by the Folin-Ciocalteu method as previously described [23], using pyrocatechol as the standard and five concentrations levels (2, 5.5, 11, 22, and 33 *μ*g/mL) for the calibration curve. DMSO was used as the solvent. The assay was carried out in a 96-well microplate. The reaction mixture contained 35 *μ*L of Folin-Ciocalteu reagent and 35 *μ*L of oil or different concentration of the standard solution or just solvent (blank). After 5 min of incubation, 35 *μ*L of sodium carbonate solution at 20 mg/mL was added and the mixture was placed in the dark for 2 h at room temperature. The absorbances were read at 760 nm in a microplate reader (Quant, BioTek Instruments Inc.). The results were expressed as pyrocatechol equivalent (g per mL of oil). After a linear regression analysis, the coefficient of determination for the standard curve was found to be 0.9982.

### 2.6. Mutagenicity Assay (Ames Test)

The mutagenicity reverse mutation test was carried out to investigate the potential of the *C. guianensis* oils to induce genetic mutation in the *Salmonella enterica* serovar Typhimurium *(S. typhimurium)* TA97, TA98, TA100, TA102, and TA1535 strains. The test was carried out according to the preincubation method, both in the absence and presence of a metabolic activation system (4% S9 mix, Aroclor-preinduced, from Moltox Inc., USA). The negative control was 10% DMSO while known mutagens were used as the positive control substances. The positive controls without the S9 mix were as follows: 4-nitroquinoline 1-oxide (4-NQO) (5 *μ*g per plate) for TA97 and TA98; sodium azide (SA) (10 mg per plate) for TA100; mitomycin C (MMC) (1 *μ*g per plate) for TA102; and methyl methanesulfonate (MMS) (200 *μ*g per plate) for TA104. The positive controls with the S9 mix were 2-amineanthracene (2-AA) (10.0 *μ*g per plate) for TA97, TA98, and TA1535 and benzo(*α*)pyrene (B[*α*]P) (50.0 *μ*g per plate) for TA100 and TA102. A dose-finding test was carried out with and without the metabolic activation system (S9 mix) for each tester strain. A total of eight concentrations diluted in DMSO were tested from 0 to 100 *μ*L/mL [24].

For the assays without metabolic activation, 0.5 mL of 0.1 mol/L sodium-phosphate buffer (pH 7.4) was added, and for the assays in the presence of metabolic activation, 0.5 mL of the S9 mix was mixed with 0.1 mL culture medium (2 × 10^9^ cells/mL) plus 0.1 mL of each compound solution (0.02 to 100 *μ*L/plate). The mixtures were incubated in a shaker at 37°C (preincubation). After 30 min preincubation protected from the light, the mixture was added to and mixed with 2 mL top agar containing 0.05 mmol/L L-histidine and D-biotin for the *S. typhimurium* strains. Each of these was then spread on a minimum glucose agar plate. After the top agar had solidified, the plates were incubated at 37°C for 60–72 h. Each tester strain was assayed in triplicate, and the number of revertant colonies was counted for each tester strain and treatment group [25]. The results were judged to be positive when the average number of revertant colonies in each treated group increased with increase in the compound concentration, reaching at least twice the number in the negative control group [24].

In order to determine the cytotoxic effects, after 30 min preincubation, the assay mixtures were diluted (1 : 10^5^) in 0.9% NaCl (*w/v*) and a suitable aliquot of the final dilution (100 *μ*L) of this suspension was plated on a nutrient agar (0.8% bacto nutrient broth (Difco), 0.5% NaCl, and 1.5% agar). The plates were then incubated at 37°C for 24 h, and the colonies were counted. All the experiments were done in triplicate and repeated at least twice. Statistical differences between the groups were analyzed by a one-way ANOVA (*P* < 0.05) and Tukey's post hoc test [24].

### 2.7. Eukaryotic Cell Cultures

Chinese Hamster Ovary (CHO-K1) and mouse macrophages (RAW264.7) cells obtained from the American Type Culture Collection (Manassas, VA) were cultured in Eagle's medium (MEM, GIBCO®, USA) containing 10% fetal bovine serum (FBS) plus 100 *μ*g/mL streptomycin and 100 *μ*g/mL penicillin at 37°C in a 5% CO_2_ atmosphere. Logarithmic phase cells were used in all the experiments [26].

### 2.8. Eukaryotic Cell Viability (WST-1)

Fresh RAW264.7 and CHO-K1 cells were seeded at a density of 1 × 10^4^ cells/well. The water-soluble tetrazolium salt assay (WST-1, Roche) (4-[3-(4-iodophenyl)-2-(4-nitrophenyl)-2H-5-tetrazolio]-1,3-benzene disulfonate) (Roche Co., South San Francisco, CA) was used to determine the number of viable cells after 3 and 24 h of exposure to the oils (1–3) (10 to 100 *μ*L/mL, diluted in DMSO). This salt is reduced by mitochondrial dehydrogenases in living cells, yielding a yellow product that is soluble in the cell culture medium. Briefly, after treatment, the culture medium was replaced by 90 *μ*L fresh culture medium and 10 *μ*L WST-1 reagent and incubated at 37°C with 5% CO_2_ for 3 h. The absorbance was then measured at 440 nm according to the kit protocol [27]. The intensity of the yellow color in the negative control (DMSO 1%) wells was designated as 100% viability, and all further comparisons were based on this reference level to determine the lethal concentration (LC_50_) for 50% of cultured cells. Statistical differences between the groups were analyzed by one-way ANOVA (*P* < 0.01) and Tukey's post hoc test.

### 2.9. Micronucleus Assay in the Cell Cultures (MN)

Fresh RAW264.7 and CHO-K1 cells were seeded at a density of 1 × 10^5^ cells/mL in 24-well plates (1 mL/per well). The oils (1–3) were then added to the medium to final concentrations from 10 to 100 *μ*L/mL diluted in DMSO, and the incubation continued for 3 h or 24 h. DMSO (1%) was used as the negative control, and N-methyl-N′-nitro-N-nitrosoguanidine (MMNG, 500 *μ*M) for RAW264.7 and MMC (5 *μ*M) for CHO-K1 were the positive controls. After exposure to the compounds, the cells were incubated for a further 24 h under growth conditions before quantification of the micronuclei and cytotoxicity. The cytogenetic studies were carried out in triplicate as described previously [28]. In order to determine the mitotic index and the number of cells with micronuclei, the medium was replaced by a cold methanol-glacial acetic acid (3 : 1) fixative for 30 min, and the cells were then rinsed with distilled water for 2 min and air-dried. The fixed cells were stained with 4,6-diamidino-2-phenylindole (DAPI) (0.2 pg/mL) dissolved in McIlvaine buffer (0.1 M citric acid plus 0.2 M Na_2_HPO_4_, pH 7.0) for 60 min, washed with McIlvaine buffer for 5 min, briefly rinsed with distilled water, and mounted in glycerol. To determine the mitotic index and the number of cells with micronuclei, 1000 cells per well (3000 cells per concentration) were analyzed under a fluorescence microscope. Cells that glowed brightly and had homogenous nuclei were considered as having normal phenotypic morphology. Apoptotic nuclei were identified by the condensed chromatin at the periphery of the nuclear membrane or by fragmented nuclear body morphology. Necrotic cells presented chromatin forms with irregularly shaped aggregates, a pyknotic nucleus (shrunken and darkly stained) and cell membrane disruption, with cellular debris spilling into the extracellular milieu. The percentage of viable cells was evaluated discounting apoptotic and necrotic cells. Statistical differences between the groups were analyzed by one-way ANOVA (*P* < 0.01) and Tukey's post hoc test.

## 3. Results

### 3.1. Extractions and Oil Yields

The seed moisture was around 30%. The oil yields (dry weight basis) for pressing the dried seeds without autoclaving (oil 1) and with autoclaving (oil 2) were 14.85% and 20.62%, respectively. The Soxhlet extraction yield (oil 3) was 61.50%.

### 3.2. Fatty Acid Composition

Table 1 presents the fatty acid compositions of the *C. guianensis* seed oils. There were no significant differences (*P* > 0.05) between the oils evaluated. The main fatty acids were oleic acid (C18:1), palmitic acid (C16:0), linoleic acid (C18:2), and stearic acid (C18:0). The monounsaturated fatty acids comprised about 50% of the total, while the saturated and polyunsaturated fatty acid contents were around 38 and 11%, respectively. The iodine and saponification values were calculated and presented in Table 1.

### 3.3. Physicochemical Property Profiles

Table 2 shows some physicochemical aspects of the oils. No differences were observed between the three oils in the following aspects: acidity, peroxides, refraction, density, and phenolic compound. On the other hand, oil 1 showed a higher scavenging activity against the DPPH radical when compared to oils 2 and 3.

### 3.4. *Salmonella*/Microsome

The results of the *Salmonella*/microsome assay (Table 3) showed that only oil 2 had mutagenic activity in the presence of exogenous metabolism, because the highest MI was 2.2, although it is possible to observe a gradual increase of the MI values with no statistical significance, mainly for oils 2 and 3 after metabolic activation. All of them induced decreased survival for most conditions, suggesting cytotoxicity with respect to different strains of *S. typhimurium*. Bacterial cytotoxicity was more evident for TA1535 with oil 1 (as from 0.1 *μ*L/plate) in the absence of S9, followed by oil 2 (as from 6.75 and 12.5 *μ*L/plate) in the absence and presence of S9, respectively, and oil 3 (as from 12.5 *μ*L/plate) in the absence and presence of S9.

### 3.5. WST-1 Assay

The cytotoxic effects in the cell lines presented different profiles. After 3 h of exposure, oil 1 was the least toxic for the CHO-K1 and RAW264.7 cells (*P* < 0.01), followed by oil 3 and oil 2 (Table 4). After 24 h of exposure, the same behavior was observed for the CHO-K1 cells, but for RAW264.7, oil 3 had the highest LC_50_ (62.91 ± 7.35), followed by oil 1 (48.10 ± 3.11) and oil 2 (46.67 ± 8.32). Finally, when comparing the two cell lines, it can be seen that the cytotoxic effects were higher in the presence of CHO-Kl.

### 3.6. Micronucleus Assay

**Figure 1:**
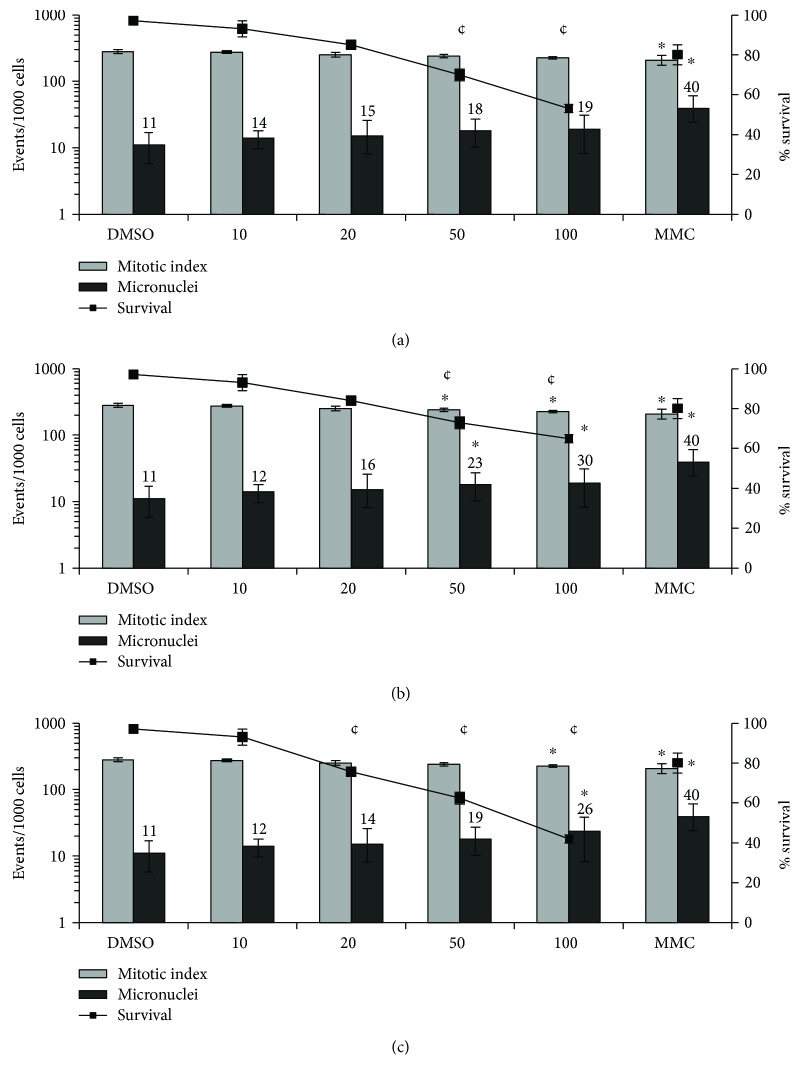
Micronucleus formation, mitotic indexes, and survival rate of CHO-K1 cells after 3 h of exposure. CHO-K1 cells were exposed to (a) oil 1, (b) oil 2, and (c) oil 3. 3000 cells were scored per treatment for each experiment. *n* = 3; ^∗^*P* < 0.001on micronucleus formation or cell division and ¢ = cytotoxic (*P* < 0.01) one-way ANOVA followed by Tukey's post hoc test.

Figure 1 shows the results for the MN assay using CHO-K1 cells. Oil 1 did not reduce the mitotic index and was not able to induce MN formation in ovary cells (Figure 1(a)). Oil 2 induced ovarian MN increasing on 2.1 times more at 50 *μ*L/mL and 2.7 times more at 100 *μ*L/mL exposures in relation to the negative control. Oil 2 also significantly reduced the mitotic index at 100 *μ*L/mL (Figure 1(b)). Oil 3 reduced the mitotic index as from 50 *μ*L/mL and increased MN formation at 100 *μ*L/mL (2.4-fold) (Figure 1(c)). The three oils induced a significant (*P* < 0.01) reduction in the survival rates.

**Figure 2:**
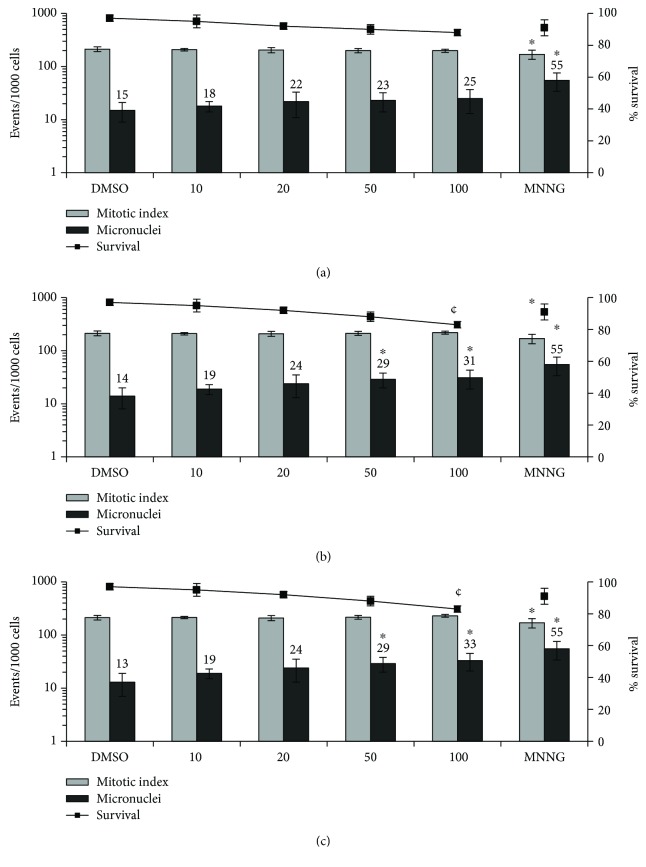
Micronucleus formation, mitotic indexes, and survival rate of RAW264.7 cells after 3 h of exposure. RAW264.7 cells were exposed to (a) oil 1, (b) oil 2, and (c) oil 3. 3000 cells were scored per treatment for each experiment. *n* = 3; ^∗^*P* < 0.001 on micronucleus formation or cell division and ¢ = cytotoxic (*P* < 0.01) one-way ANOVA followed by Tukey's post hoc test.

Using RAW264.7 macrophages (Figure 2), oil 1 did not cause DNA damage or delay in the cell cycle (Figure 2(a)). Oil 2 (Figure 2(b)) induced significant MN induction at 50 *μ*L/mL (2.1-fold) and 100 *μ*L/mL (2.2-fold), and oil 3 (Figure 2(c)) also induced significant MN increasing at exposures of 50 *μ*L/mL (2.2-fold) and 100 *μ*L/mL (2.5-fold). None of the oils induced cytostatic effects on macrophages. Both oils 2 and 3 presented significant (*P* < 0.01) cytotoxic response at 100 *μ*L/mL.

## 4. Discussion

The use of natural products in traditional Brazilian phytomedicine is widely accepted and prescribed, mainly in the poorest areas, such as the Amazon and Northeastern regions. Thus, the pharmaceutical potential of Brazilian herbs must be considered in drug discovery studies [29]. Contrary to allopathy, the traditional usage of medicinal plants is apparently considered to be safe and hence the toxicity of traditional herbal medicines has not been totally evaluated in most cases, although medicinal plants can be extremely harmful to human health. Studies have revealed that some plants frequently used in folk medicine are potentially genotoxic [30–32]. It is thus truly relevant to screen the genotoxicity during the preclinical evaluation of herbal extracts or substances, in order to verify their mutagenic potential for both safety and economic concerns, since plants are widely used in folk medicine and can be a resource for the development of new drugs [33].

The drying process was required in the oil extraction processes by both solvent extraction (Soxhlet) and screw pressing. The expeller pressing yields were similar to the results reported by Souza et al. [14]. Autoclaving before drying improved the oil yield from ~15% to 20% while the Soxhlet extraction yielded 61.5%, suggesting that the extraction process using petroleum ether at 30–60°C with no further heat treatment is the more efficient in terms of yield than the pressing, although the raw material is difficult to handle due to its rubbery texture. Under the conditions used, the Soxhlet extraction allowed for the extraction of all the oil besides other petroleum ether-soluble compounds. The screw press or hydraulic press usually used for low moisture content seeds and nuts recovers around 50 to 90% of the oil, depending on the pretreatment carried out, the capacity and design characteristics of the equipment, the temperature, and the seed moisture content, but remarkable variations were observed regarding the composition of the raw material.

The extraction methods using heating processes to improve the extraction yield have been used before in traditional use of *C. guianensis* oils for medicinal use in the Amazon area [13]. In the present work, autoclaving improved the extraction performance indicating that the thermal treatment is required to denature the cellular structure for oil removal. However, lower temperatures used in the Soxhlet extraction compared to extraction process by autoclaving increase the extraction yield.

It has been reported that *C. guianensis* seed oils contain the myristic, palmitic, oleic, linoleic, stearic, and arachidic fatty acids, tetraterpenoids [34], and flavane [35]. Here, we determine the main fatty acids found in the andiroba oil such as oleic (50%), palmitic (28%), stearic (9%), and linoleic (10%). The linolenic (C18:3) and palmitoleic (C16:1) acids were detected up to 1.4 and 0.9%, respectively. Some minor fatty acids such as C14:0, C17:0, C20:1, C20:1, C22:0, and C24:0 were detected at values below 0.4%. The acidity or free fatty acid content and peroxide value of the andiroba oil were very low and statically similar results (*P* > 0.05), as shown in Table 2, which is unusual for this oil since oil acidity of 30% has been observed using the traditional process, which allows for enzyme action and fermentation.

The seeds were collected and frozen before drying followed by the oil extraction process, hence avoiding enzyme activity related to triacylglycerol hydrolysis and the oxidative process. Nevertheless, the cell wall degrading enzymes may release compounds besides fatty acids which show biological activity. The possible differences in the biological activity of andiroba oil due to the extraction process have not been clearly established [12, 13]. In addition, there were no statistical differences between the three oils regarding the refraction and density suggesting that the differentiated extraction processes did not alter such physicochemical aspects.

In spite of the fact that oil 1 presented a higher phenolic content, no statistical differences were observed between the three oils in the phenolic compound determination (Table 2), suggesting that the extractable phenolic compounds are not associated with free radical scavengers, since oil 1 showed greater scavenging activity against the DPPH radical. Thus, it appears that the absence of high temperature for extraction associated with the shortest processing time prevented possible degradation of active components in the extract, which led to the greatest scavenging activities. Thermal treatment, as frying or boiling, can modify the composition of other vegetable oils, as crude palm oil, soybean oil, canola oil, flaxseed oil, and sunflower oil, increasing the oxidization status and enhancing the deleterious effects on human and animal health after consumption [36–40]. Milhomem-Paixão et al. [41] evaluated several andiroba oil samples and showed IC_50_ values for DPPH scavenging activity similar to those presented here. Probably the extraction that we performed without heat contains higher amount of antioxidant compounds such as phenolic compounds extracted with water present in the material (Table 2) or volatile compounds in the seeds. Therefore, the biological properties of the oils do not appear to be associated with the presence of phenolic compounds.

In addition to fatty acids, limonoids, triterpenes, steroids, coumarins, flavonoids, and diglycerides have been isolated from several parts of *C. guianensis* [42]. *C. guianensis* seed oils are rich source in limonoids, highly oxygenated tetranortriterpenoid compounds, that are reported to present several biological activities, such as antifungal, bactericidal, antifeedant, antimalarial, antiviral, anti-inflammatory, and growth-regulator on insects [43, 44], besides several medicinal effects in animals and humans [2]. Ambrozin et al. [42] identified seven limonoids in oil sample of *C. guianensis*: 17*β*-hydroxyazadiradione, gedunin, 6*α*-acetoxygedunin, 7-deacetoxy-7-oxogedunin, 1,2-dihydro-3*β*-hydroxy-7-deacetoxy-7-oxogedunin, methyl angolensate, and xyloccensin K, by various chromatographic techniques. These bioactive compounds are responsible for the therapeutic effects of the oil [2]. In agreement with the literature, the present study demonstrated the presence of monounsaturated, polyunsaturated, and saturated fatty acids.

In the present study, the *Salmonella*/microsome and micronucleus tests were used to screen the *C. guianensis* seed oils for genotoxicity. As shown here, except for oil 2, in the presence of metabolic activation for TA100, no mutagenic activity by frameshift was shown (TA97 and TA98 strains) and base pair substitution mutations (TA100, TA102, and TA1535 strains) were detected for all three oils, considering that the criterion for mutagenicity is indicated for MI higher than or equal to 2, although it is possible to observe a gradual increase of the MI values with no statistical significance, mainly for oils 2 and 3 after metabolic activation. On the other hand, all oils induced cytotoxic effects. These effects may be related to the chemical composition of the oils. So, cytotoxic effects observed in the present study may be related to the bactericidal activity and can be attributed to the limonoids [2].

The WST-1 assay is based on the capacity of the cellular mitochondrial dehydrogenase enzyme in living cells to reduce the water-soluble tetrazolium salt into the yellow-colored formazan dye, which is insoluble in water. The amount of formazan produced is directly proportional to the cell number in a range of cell lines. Thus, this assay relies on the mitochondrial activity of the cells and represents a parameter for their metabolic activity [45]. The WST-1 assay showed that the cell viabilities of CHO-Kl and RAW264.7 were affected by oils 1, 2, and 3 after 3 and 24 h exposure, inducing cell death. It is worthwhile noting that after 3 h of exposure, the cytotoxicity was higher for CHO-Kl (Table 4). Considering the induction of increased cell death in the two cell lines, the MN assay was carried out after only 3 h treatment. Except for oil 1, andiroba oils induce clastogenic and aneugenic effects in Chinese hamster ovaries (Figure 1) and macrophages (Figure 2). These findings indicate that the highest extraction temperature associated with the longest processing time leads to an increase in compounds which induce DNA damage. Oils 2 and 3 also induced a decrease in the mitotic index, but only for CHO-Kl (Figure 1), suggesting cell cycle arrest. Some studies have shown that gedunin, a tetranortriterpenoid present in *C. guianensis* oil, inhibits the proliferation of cancer cells, mainly by the inhibition of cell cycle arrest in different tumor cell lines, including ovarian cancer, via the induction of apoptosis by the cleavage of cochaperone p23 [46]. In addition, corroborating the results obtained with respect to cytotoxicity for CHO-Kl, the three oils induced a reduction in the survival rates. According to Llana-Ruiz-Cabello et al. [47], considering the commercial interest in vegetable oils and their components, the sources of exposure are substantially enhanced. Thus, data concerning the genotoxicity of these substances are needed in order to better understanding these safety profiles.

## 5. Conclusion

Overall, we conclude that, related to fatty acid composition and physicochemical property, there were no significant differences between the three oils. In this way, differentiated extraction processes did not alter such composition or property. Significant levels of cytotoxicity in bacterial and eukaryotic models were induced by the three oils, suggesting that these effects may be related to its chemical composition. Oil 1 obtained from *C. guianensis* seeds, which was extracted without the use of high temperatures, showed to be the safest for use and the most promising product as compared to the other oils, since it was not mutagenic or genotoxic and showed a higher scavenging activity against the DPPH radical, unlike that observed for oils 2 and 3. The data discussed in this paper contributed to the knowledge that processing the *C. guianensis* seeds at high temperatures increases the risk of adverse genotoxic effects and decreases the scavenging activities of crabwood seed oil.

## Figures and Tables

**Table 1:** Fatty acid composition (g/100 g) of *Carapa guianensis* oils.

Fatty acid	*Carapa guianensis* oils (mean ± SD)		
	Oil 1	Oil 2	Oil 3
C14:0 (myristic)	0.05 ± 0.000	0.04 ± 0.001	0.05 ± 0.000
C16:0 (palmitic)	27.71 ± 0.016	27.50 ± 0,039	28.33 ± 0.123
C16:1 (palmitoleic)	0.90 ± 0.001	0.89 ± 0.001	0.94 ± 0.005
C17:0 (margaric)	0.12 ± 0.001	0.12 ± 0.001	0.13 ± 0.001
C18:0 (stearic)	9.34 ± 0.007	9.52 ± 0.002	9.09 ± 0.025
C18:1 (oleic)	49.90 ± 0.012	49.71 ± 0.025	49.56 ± 0.074
C18:2 (linoleic)	9.58 ± 0.010	9.82 ± 0.011	9.62 ± 0.013
C18:3 (linonelic)	1.43 ± 0.002	1.43 ± 0.003	1.38 ± 0.011
C20:0 (arachidic)	0.26 ± 0.002	0.27 ± 0.001	0.23 ± 0.001
C20:1 (gondoic)	0.13 ± 0.003	0.13 ± 0.003	0.13 ± 0.004
C22:0 (behenic)	0.36 ± 0.001	0.35 ± 0.003	0.35 ± 0.004
C24:0 (lignoceric)	0.22 ± 0.004	0.22 ± 0.002	0.21 ± 0.002
Iodine value (g I_2_/100 g)	196.6	196.6	196.6
Saponification value (mg KOH/g)	59.5	59.7	59.3

SD: standard deviation. No significant differences: *P* > 0.05 ANOVA and Tukey's between the oils.

**Table 2:** Physicochemical aspects of *Carapa guianensis* oils.

	Oil 1	Oil 2	Oil 3
Acidity (% oleic acid)	0.30^a^	0.28^a^	0.24^a^
Peroxides (meq/kg)	0.97^a^	0^a^	0^a^
Refraction (nD 40°C)	1.4595^a^	1.4603^a^	1.4593^a^
Density (g/cm^3^)	0.9174^a^	0.9183^a^	0.9169^a^
EC_50_ scavenging of DPPH^+^ (*μ*L/mL)	89.07 ± 3.67^a^	>200^b^	>200^b^
Phenolic content (mg/g of catechol)	10.34 ± 0.04^a^	9.50 ± 0.02^a^	9.00 ± 0.03^a^

Values followed by different letters on the same line differ statistically amongst themselves according to ANOVA and the Tukey's test (*P* < 0.05). CE50: half-maximal effective concentration; DPPH: 2,2-diphenyl-1-picrylhydrazyl. Values of two independent assays.

**Table 3:** Mean values ± SD (MI) of revertant *His^+^* colonies of *Salmonella enterica* serovar Typhimurium strains used in *Salmonella*/microssome assay after coincubation with *Carapa guianensis* oils.

		*μ*L/plate	TA97	TA98	TA100	TA102	TA1535
Oil 1	−S9	0	67 ± 1 (1.0)	41 ± 9 (1.0)	118 ± 28 (1.0)	428 ± 16 (1.0)	14 ± 5 (1.0)
	−S9	0.02	92 ± 8 (1.4)	41 ± 12 (1.0)	168 ± 18 (1.4)	429 ± 9 (1.0)	16 ± 2 (1.1)
	−S9	0.1	89 ± 13 (1.3)	36 ± 7 (0.9)	180 ± 13 (1.5)	443 ± 39 (1.0)	Cytotoxic
	−S9	0.2	73 ± 10 (1.1)	45 ± 1 (1.1)	190 ± 19 (1.6)	462 ± 12 (1.1)	—
	−S9	1	82 ± 9 (1.2)	41 ± 4 (1.0)	172 ± 28 (1.5)	Cytotoxic	—
	−S9	2	Cytotoxic	40 ± 6 (1.0)	148 ± 18 (1.3)	—	—
	−S9	3.38	—	Cytotoxic	Cytotoxic	—	—
	+S9	0	180 ± 35 (1.0)	49 ± 7 (1.0)	169 ± 1 (1.0)	534 ± 73 (1.0)	30 ± 4 (1.0)
	+S9	0.02	185 ± 28 (1.0)	54 ± 6 (1.1)	171 ± 15 (1.0)	554 ± 34 (1.0)	31 ± 11 (1.0)
	+S9	0.1	187 ± 27 (1.0)	61 ± 20 (1.2)	179 ± 10 (1.1)	615 ± 41 (1.2)	33 ± 4 (1.1)
	+S9	0.2	166 ± 21 (0.9)	48 ± 3 (1.0)	181 ± 45 (1.1)	639 ± 39 (1.2)	36 ± 2 (1.2)
	+S9	1	150 ± 18 (0.8)	47 ± 4 (1.0)	172 ± 21 (1.0)	710 ± 70 (1.3)	36 ± 6 (1.2)
	+S9	2	139 ± 35 (0.8)	56 ± 6 (1.1)	183 ± 7 (1.1)	798 ± 10 (1.5)	44 ± 8 (1.5)
	+S9	3.38	Cytotoxic	Cytotoxic	Cytotoxic	Cytotoxic	Cytotoxic

Oil 2	−S9	0	93 ± 1 (1.0)	37 ± 3 (1.0)	146 ± 32 (1.0)	358 ± 2 (1.0)	12 ± 4 (1.0)
	−S9	2	93 ± 2 (1.0)	37 ± 6 (1.0)	148 ± 11 (1.0)	311 ± 4 (0.9)	13 ± 2 (1.1)
	−S9	3.38	93 ± 10 (1.0)	38 ± 4 (1.0)	152 ± 19 (1.0)	268 ± 33 (0.8)	13 ± 3 (1.1)
	−S9	6.75	104 ± 10 (1.1)	40 ± 6 (1.1)	164 ± 16 (1.1)	289 ± 55 (0.8)	Cytotoxic
	−S9	12.5	104 ± 15 (1.1)	33 ± 5 (0.9)	171 ± 21 (1.2)	283 ± 105 (0.8)	—
	−S9	25	99 ± 6 (1.0)	47 ± 4 (1.3)	176 ± 14 (1.2)	291 ± 44 (0.8)	—
	−S9	50	96 ± 7 (1.0)	41 ± 3 (1.1)	179 ± 17 (1.2)	280 ± 50 (0.8)	—
	−S9	100	93 ± 10 (1.0)	31 ± 7 (0.8)	Cytotoxic	388 ± 50 (1.1)	—
	+S9	0	135 ± 8 (1.0)	23 ± 3 (1.0)	173 ± 20 (1.0)	354 ± 38 (1.0)	14 ± 3 (1.0)
	+S9	2	139 ± 7 (1.0)	27 ± 5 (1.2)	189 ± 18 (1.1)	365 ± 37 (1.0)	15 ± 3 (1.1)
	+S9	3.38	133 ± 11 (1.0)	28 ± 1 (1.2)	188 ± 13 (1.1)	386 ± 42 (1.1)	16 ± 4 (1.1)
	+S9	6.75	186 ± 15 (1.4)	29 ± 5 (1.3)	228 ± 16 (1.3)	403 ± 19 (1.1)	21 ± 7 (1.5)
	+S9	12.5	191 ± 12 (1.4)	30 ± 5 (1.3)	260 ± 12 (1.5)	396 ± 31 (1.1)	Cytotoxic
	+S9	25	194 ± 13 (1.4)	39 ± 1 (1.7)	306 ± 10 (1.8)	372 ± 40 (1.0)	—
	+S9	50	208 ± 16 (1.5)	42 ± 4 (1.8)	333 ± 15 (1.9)	399 ± 18 (1.0)	—
	+S9	100	215 ± 8 (1.6)	Cytotoxic	383 ± 20 (2.2)^∗^	454 ± 32 (1.3)	—

Oil 3	−S9	0	73 ± 3 (1.0)	31 ± 9 (1.0)	132 ± 11 (1.0)	263 ± 9 (1.0)	11 ± 4 (1.0)
	−S9	2	73 ± 3 (1.0)	31 ± 3 (1.0)	132 ± 10 (1.0)	260 ± 7 (1.0)	12 ± 4 (1.1)
	−S9	3.38	77 ± 16 (1.1)	29 ± 11 (1.0)	131 ± 18 (1.0)	268 ± 31 (1.0)	12 ± 1 (1.1)
	−S9	6.75	76 ± 9 (1.0)	33 ± 4 (1.1)	138 ± 17 (1.0)	289 ± 6 (1.1)	13 ± 1 (1.2)
	−S9	12.5	75 ± 4 (1.0)	35 ± 2 (1.1)	129 ± 29 (1.0)	259 ± 22 (1.0)	Cytotoxic
	−S9	25	91 ± 6 (1.2)	31 ± 3 (1.0)	119 ± 12 (0.9)	259 ± 22 (1.0)	—
	−S9	50	93 ± 5 (1.3)	31 ± 4 (1.0)	130 ± 27 (1.0)	365 ± 26 (1.4)	—
	−S9	100	94 ± 13 (1.3)	30 ± 5 (1.0)	108 ± 6 (0.8)	388 ± 18 (1.5)	—
	+S9	0	145 ± 9 (1.0)	29 ± 4 (1.0)	193 ± 15 (1.0)	267 ± 22 (1.0)	11 ± 2 (1.0)
	+S9	2	143 ± 7 (1.0)	31 ± 5 (1.1)	199 ± 13 (1.0)	280 ± 18 (1.0)	15 ± 1 (1.4)
	+S9	3.38	151 ± 11 (1.0)	32 ± 2 (1.1)	208 ± 9 (1.1)	299 ± 25 (1.1)	17 ± 4 (1.5)
	+S9	6.75	Cytotoxic	35 ± 3 (1.2)	Cytotoxic	318 ± 16 (1.2)	19 ± 6 (1.7)
	+S9	12.5	—	40 ± 6 (1.4)	—	344 ± 22 (1.3)	Cytotoxic
	+S9	25	—	44 ± 8 (1.5)	—	372 ± 21 (1.4)	—
	+S9	50	—	49 ± 3 (1.7)	—	Cytotoxic	—
	+S9	100	—	Cytotoxic	—	—	—

SD: standard deviation; −S9: absence of metabolic activation; +S9: presence of metabolic activation; MI: mutagenicity index; ^∗^Difference of negative control, one-way ANOVA followed by Tukey's post hoc test (*P* < 0.05). Each sample was assayed until a cytotoxic response (survival < 70%); positive controls without S9: 4-NQO (1.0 *μ*g/pl.) for TA97, 286 ± 17 revertants, TA98 120 ± 10 revertants, and TA1535 746 ± 58 revertants; AS (1.0 *μ*g/pl.) for TA100, 607 ± 56 revertants; MMC (0.5 *μ*g/pl.) for TA102; with S9: 2AA (1.0 *μ*g/pl.) for TA97, 587 ± 11 revertants for TA98, 305 ± 1 revertants and for TA100, 1436 ± 40 revertants; B[a]P (50 *μ*g/pl.) for TA102, 1448 ± 79 revertants and for TA1535 111 ± 10 revertants.

**Table 4:** Cytotoxicity (LC_50_) of *Carapa guianensis* oils after 3 h and 24 h of treatment in RAW264.7 and CHO-K1 cells.

	LC_50_ (*μ*L/mL)			
	CHO-K1		RAW264.7	
	3 h	24 h	3 h	24 h
Oil 1	73.81 ± 2.32	40.48 ± 5.13	93.45 ± 6.82	48.10 ± 3.11
Oil 2	45.24 ± 7.67^a^	26.19 ± 6.67^a^	80.13 ± 6.14	46.67 ± 8.32^a^
Oil 3	66.67 ± 3.18^b^	28.57 ± 3.33^a^	87.36 ± 9.41	62.91 ± 7.35^a,b^

LC_50_: lethal concentration. ^a^*P* > 0.01 versus oil 1 and ^b^*P* > 0.01 versus oil 2; *n* = 3; one-way ANOVA followed by Tukey's post hoc test.
